# Editorial Notes

**Published:** 1884-07

**Authors:** 


### Editorial Notes.

A fashionable antiseptic in all the hospitals of New York city is the solution of bichloride of mercury, one part to fifteen hundred or two thousand of water.

During the year 1883, 298,153 pounds of manufactured opium for smoking purposes were imported into this country, on which the Government received, at $6 a pound, a customs revenue of nearly $200,000,000. The query is, what becomes of all of this opium, and by whom is it consumed ?

THE value of tongaline in the treatment of neuralgia and rheumatism is established by many careful observers in this country as well as by Ringer and Murrell of London, England. The use of the preparations of opium in neuralgia, especially, is open to the great danger to the patient of the establishment of the "opium habit." An agent preventing this, and which is almost a specific in the acute forms of neuralgia, is to be welcomed and employed by every physician.

WE desire to call attention to some valuable medicinal preparations which are worthy of receiving greater attention from medical men. Messrs. Wyeth & Bro. are furnishing compressed pills of bisulphate of quinine, which are readily soluble and cannot fail to dissolve when swallowed. We have critically examined the method followed by Wyeth & Bro. in preparing their compressed pills and other pharmaceuticals, with the result of decidedly increasing our previous high opinion of their preparations.

THE hot weather of this and the coming month will bring with it the usual proportion of "pain and trouble," especially among infants and children, due to digestive disturbances. In the majority of these cases, after the proper regulation of the food, we have found the use of some of the pepsin preparations of service. Lactopeptine is perhaps the best to employ; at least, we have never failed to find the preparation effective when a digestive agent was indicated. Composed as it is of pepsin, pancreatic, ptyalin, lactic and hydrochloric acids, it fairly represents the composition of the natural digestive juices, and is undoubtedly superior to pepsin alone.

